# Impaired Mitochondrial and Glycolytic Functions in Peripheral Blood Leukocytes of Alzheimer’s Disease

**DOI:** 10.1093/geroni/igaa057.395

**Published:** 2020-12-16

**Authors:** Ravindra Bharadwaj, Hayley Gibler, Sanjay Srivastava, Hena Tewari

**Affiliations:** 1 Texas Tech University Health Sciences Center, Amarillo, Texas, United States; 2 Tecas Tech HSC AMA, Amarillo, United States; 3 Texas Tech HSC, Amarillo, United States; 4 Texas Tech HSC - AMA, Amarillo, Texas, United States

## Abstract

Recent failures of the trials targeting amyloid to treat Alzheimer’s disease (AD) are prompting scientists to explore other pathological pathways. Brains of AD patients have been noted to have impaired mitochondrial function. It has not yet been determined if AD is caused by a systemic defect in cellular bioenergetics. To determine the cellular bioenergetics, we compared the Oxygen Consumption Rate (OCR – indicating oxygen dependent respiration) and Extra Cellular Acidification Rate (ECAR – indicating glycolytic function) in leukocytes of collected blood samples of Alzheimer’s and non-dementia patients. Methods: After IRB approval and consents, blood samples from each clinically diagnosed Alzheimer’s and age matched normal subjects were collected. Immediately after collection the blood samples were analyzed using Agilent Seahorse XFe/XF Analyzer as per protocol by manufacturer. Results: Impaired mitochondrial and glycolytic functions were noted in Alzheimer’s patients as compared to normal subjects. OCR was significantly lower in Alzheimer’s patients. A lower rate of respiration was noted both at basal as well as maximal respiration. Reduced spare respiration capacity was also noted in response to the stressors. Similarly reduced ECAR and reduced glycolytic reserve was also noted in Alzheimer’s patients, indicating impaired oxygen independent mitochondrial respiration. Discussion: This pilot study demonstrates that there is an impaired mitochondrial and glycolytic function in the peripheral blood cells. This indicates towards a systemic nature of the disease and a potential future bio-marker. Further studies should be planned in this direction.

